# Significant Microvascular Abnormalities Present in Autonomic Nervous System Dysfunction: Results of a Cross-Sectional Study

**DOI:** 10.3390/biomedicines13051242

**Published:** 2025-05-20

**Authors:** Sehreen Mumtaz, Karissa Arca, Vikas Majithia, William Cheshire, David Hodge, Florentina Berianu

**Affiliations:** 1 Department of Rheumatology, Mayo Clinic Florida, Jacksonville, FL 32224, USA; majithia.vikas@mayo.edu (V.M.); berianu.florentina@mayo.edu (F.B.); 2 Department of Neurology, Mayo Clinic Arizona, Scottsdale, AZ 85259, USA; arca.karissa@mayo.edu; 3 Department of Neurology, Mayo Clinic Florida, Jacksonville, FL 32224, USA; cheshire@mayo.edu; 4 Department of Quantitative Health Sciences, Mayo Clinic Florida, Jacksonville, FL 32224, USA; hodge@mayo.edu

**Keywords:** nailfold video capillaroscopy (NVC), autonomic dysfunction (AD), dysautonomia

## Abstract

**Purpose:** The prevalence and phenotype of capillaroscopic abnormalities in patients with autonomic nervous system dysfunction have not yet been investigated. Multiorgan involvement in dysautonomia entails abnormal vasoreactivity. We aim to correlate the diagnosis of autonomic dysfunction with certain clinical manifestations, which may provide prognostic or diagnostic information using a noninvasive technique, i.e., nailfold video capillaroscopy (NVC). **Methods:** Patients with autonomic nervous system dysfunction were recruited from rheumatology and neurology clinics with voluntary NVC procedures from 31 January 2024 to 10 January 2024, and a comparison with normal controls was performed. Additional recorded information include demographics and diagnoses of autonomic dysfunction types by autonomic testing, including, but not limited to, the following: reflex screen, sweat test, Valsalva maneuver, nerve fiber density, electromyography (EMG), serology, and history of autoimmune diseases. NVC was performed on a total of 27 patients. This study was approved by the Mayo Clinic Institutional Review Board. **Results:** The autonomic dysfunction group consisted of small-fiber neuropathy (37%), orthostatic hypotension (48%), autonomic neuropathy (30%), limited autonomic neuropathy (7%), postural orthostatic tachycardia syndrome (POTS) (7%), and connective tissue disease (7%), among other types. Patients with autonomic dysfunction had statistically significant increases in microhemorrhages, dilated capillaries, and ramifications when compared to controls. **Conclusions:** Autonomic dysfunction was associated with statistically significant microvascular abnormalities compared to normal controls with a distinct NVC pattern. There was a statistically significant correlation between age and BMI with microvascular abnormalities. Here, we demonstrate the diagnostic potential of NVC in autonomic dysfunction and advocate for further study of capillary structures in autonomic dysfunction.

## 1. Introduction

The autonomic nervous system is a component of both the peripheral and central nervous system and comprises the sympathetic and parasympathetic nervous systems [1]. The sympathetic nervous system controls active responses such as increasing the heart rate and maintaining blood pressure, while the parasympathetic nervous system slows down the heart rate and stimulates digestion (for example [2]). Abnormal autonomic function is a potential risk factor for the development of vascular diseases and endothelial abnormalities, and inflammation is associated with pathological sympathetic nervous system activity [3]. Nailfold video capillaroscopy is a noninvasive method used by rheumatologists to assess the microvascular bed around nails that correlates with systemic diseases in rheumatology, such as scleroderma/dermatomyositis [4].

Given variable manifestations of autonomic dysfunctions, their diagnoses are not straightforward. Standard clinical testing of autonomic nervous system function is challenging and requires extensive and time-consuming autonomic investigations, including Valsalva, sweat test, and reflex screening with a portion of subjectivity, among other specialized testing procedures [5]. Interpretation and correlation of autonomic testing to symptoms requires caution and awareness with patients’ comorbidities and medications that may drastically alter results.

A non-invasive technique known as capillaroscopy is currently being used for rheumatologic disorders that are also linked to vascular pathology [6]. This procedure entails placing a magnifying scope on the nailbed in order to visualize the superficial capillary bed; it can pick up on abnormalities such as microhemorrhages, changes in the normal vessel architecture, or decreases in capillary density [7]. It is inexpensive, quick, and non-invasive and is known to provide objective diagnostic and prognostic benefits in patients with scleroderma and other connective tissue diseases. We aim to utilize nailfold video capillaroscopy (NVC) to assess evidence of microvascular damage and changes in capillary bed architecture, thereby correlating with the presence of autonomic dysfunction by complementing standard autonomic reflex testing. We are interested in identifying unique patient subsets with autonomic dysfunction diagnoses, which may benefit considerably from the application of NVC.

## 2. Methods

Patients with autonomic nervous system dysfunction were recruited from rheumatology and neurology clinics with voluntary NVC procedures from 31 January 2024 to 10 January 2024. NVC was performed on a total of 27 patients. This study was approved by the Mayo Clinic Institutional Review Board (IRB approval number: 23-012308; date of approval: 31 January 2024). A prospective single-center cohort study was conducted where patients with a diagnosis of autonomic dysfunction, which was confirmed by a neurologist specialist, were enrolled. Only the patients with positive abnormal autonomic dysfunction test results were included and were subsequently evaluated for NVC abnormalities.

Autonomic testing included but was not limited to reflex screen, sweat test, Valsalva maneuver, skin biopsy for nerve fiber density (if available), EMG, cardiovagal and cardio adrenergic responses, and autoimmune antibody testing. The reflex screen included the postganglionic sympathetic sudomotor testing (sweat test), heart rate response to deep breathing, Valsalva, and then head-up tilt. The testing was guided by the discretion of a neurologist/rheumatologist or another specialist as a standard of care. Patients were identified using the Advanced Text Exploratory, Mayo Data Explorer, and Epic SlicerDicer, and if they were felt to be appropriate candidates based on a valid abnormal autonomic reflex screen, they were invited for participation, which consists of a brief, non-invasive capillaroscopy procedure. At the time of the enrollment, all patients were consented and then underwent NVC.

Additional information were recorded via a chart review, which included demographics, diagnosis of autonomic dysfunction with autonomic testing results, serology as ordered by the rheumatology clinic, history of autoimmune disease, and pertinent lab results. A 1:1.3 comparison with normal controls was performed (normal cohort of 21 patients was used for a prior research study). Exclusion criteria included age <18 years, current smoker status, history of Raynaud’s phenomenon or a disease, diabetes mellitus, dialysis dependence, recent hand trauma or a manicure (within 2 weeks), history of acrocyanosis or erythromelalgia, and a previous diagnosis of bleeding disorders. Patients with abnormal autonomic reflex screens that were attributed to possible medication use were also excluded from this study.

### 2.1. Nailfold Video Capillaroscopy

Capillaroscopy was performed in a temperature-controlled environment maintained at 22° to 25° Celsius to minimize vasoconstriction. The equipment used included the Optilia Video Capillaroscopy (magnification of 200×) system, and the obtained results were analyzed by using the Optipix software (V1.7.6). Patients were in a seated position for approximately 15 min prior to the examination. A nontoxic emersion oil was applied to nailbeds, and optical images of nailbeds of digits 2 to 5 were taken. Two images per nailbed were saved, and the thumbs were excluded as per standard capillaroscopy practice. Capillaroscopy variables that were recorded included capillary density, diameter, microhemorrhages, ramifications, and disorganized capillary morphology. Density was considered reduced if there were fewer than 7 capillaries/1 mm field. Dilated capillaries were defined as greater than 20 μm but less than 50 μm in diameter. Dimensions greater than 50 μm in diameter were classified as giant capillaries. Normal capillary morphology was similar to hairpin-like loops at the last layers of capillaries within the nailbed. Abnormal morphology included bushy capillaries, ramifications, ectasia, and elongated capillaries. Microhemorrhages, macrohemorrhages, and capillary thrombi were recorded as abnormal if present. A semiquantitative scoring method was used with scores of 0 (least) to 3 (maximum) in each identified category [8]. Scoring was corroborated by two trained providers, and discrepancies in scoring were discussed and revisited until a resolution was reached.

### 2.2. Statistical Methods

Categorical variables were reported as a standard mean difference in percentages and 95% confidence intervals. Comparisons between the cases and controls were completed using Fisher’s exact tests for categorical variables. Continuous variable analyses were completed using two-sample *t*-tests. Analyses were completed using SAS version 9.4 (Cary, NC, USA). A linear regression model was used to estimate the mean change in NVC scores in association with different patient characteristics; regression coefficients and corresponding 95% confidence intervals were reported. NVC scores included capillaroscopy density, dilated capillaries, giant capillaries, microhemorrhages, ramification, and capillary disorganization. All *p*-values were two-sided without adjustments for multiple testing. Moreover, *p*-values of less than 0.05 were considered statistically significant.

## 3. Results

Baseline characteristics are depicted in Table 1. The mean age of the autonomic dysfunction (AD) group was 60.6 years with a standard deviation (SD) of 16.0 years, which was significantly higher than the control group with a mean age of 48.8 years and SD of 13.2 years (*p* = 0.005). There were no significant differences in gender and ethnicity between cases and controls. There were 15/27 (55.6%) males in the AD group and 7/21 (33.3%) males in the control group. Moreover, 24/27 (88.9%) of the AD group and 21/21 (100.0%) of the controls were not Hispanic or Latino. The autonomic dysfunction group consisted of small-fiber neuropathy 10/27 (37%), orthostatic hypotension 13/27 (48%), autonomic neuropathy 8/27 (30%), limited autonomic neuropathy 2/27 (7%), postural orthostatic tachycardia syndrome (POTS) 2/27 (7%), other central autonomic disorders 3/27 (11%), and connective tissue diseases 2/27 (7%), among other types. Sjogren’s syndrome was an autoimmune disorder in 2/2 (100%) of the connective tissue disease diagnoses.

Reduced quantitative sweat test (qsweat) was present in 19/27 (70%) of the AD group. Cardiovagal responses were reduced in 7/27 (26%) in the AD group (Table 1). Abnormal cardiovascular adrenergic responses were present in 10/27 (38%) of the AD cases. The median composite autonomic severity score (CASS) in the AD group was 3.0 with an interquartile range of (2.0, 5.0). No patients in the AD group had a flat-top response (Table 1). Electromyography was abnormal in 20/27 (77%) in the AD group (Table 1). No patients had a skin punch biopsy or a salivary gland biopsy performed. No patients had an autoimmune dysautonomia panel.

**Figure 1:**
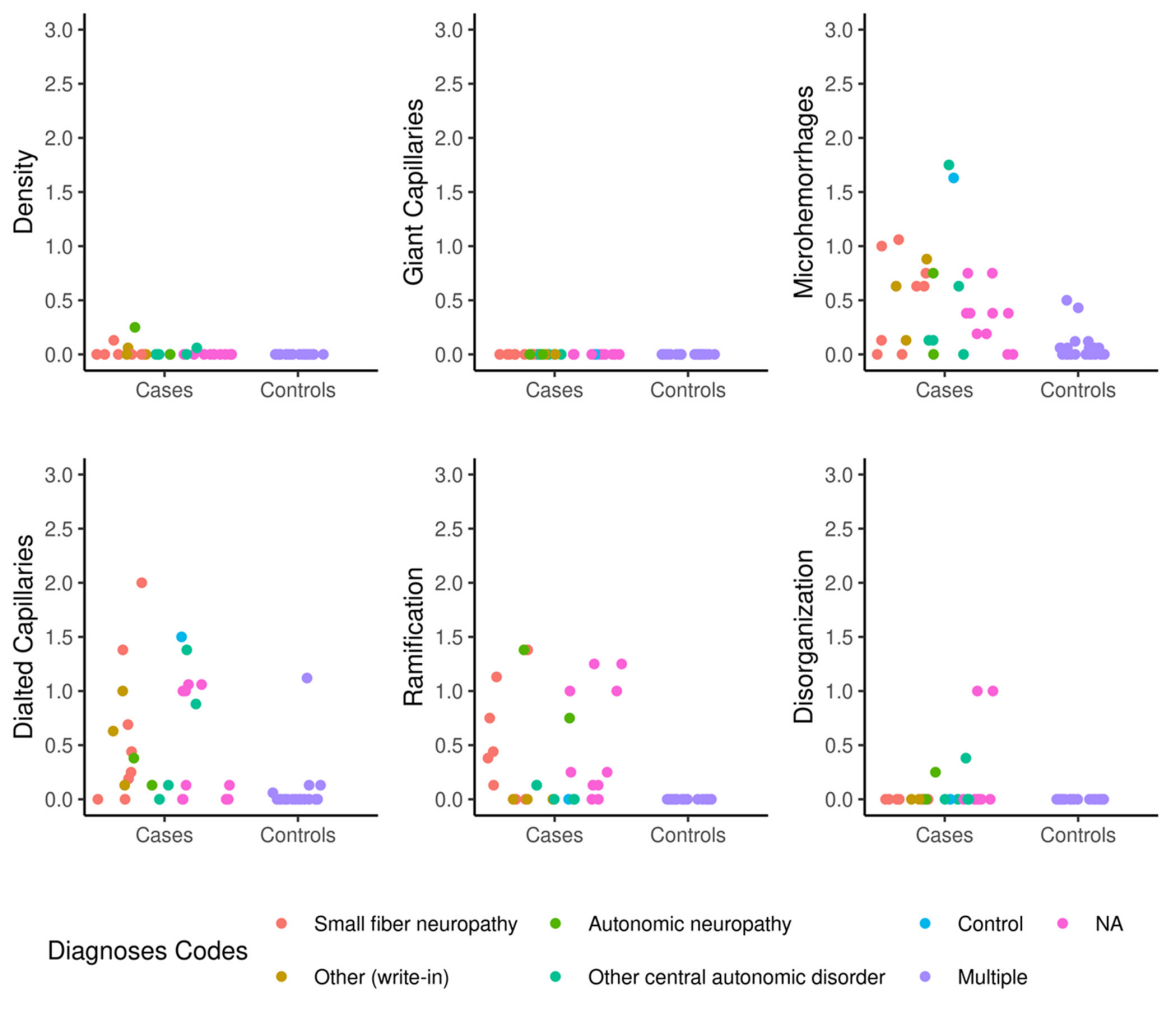

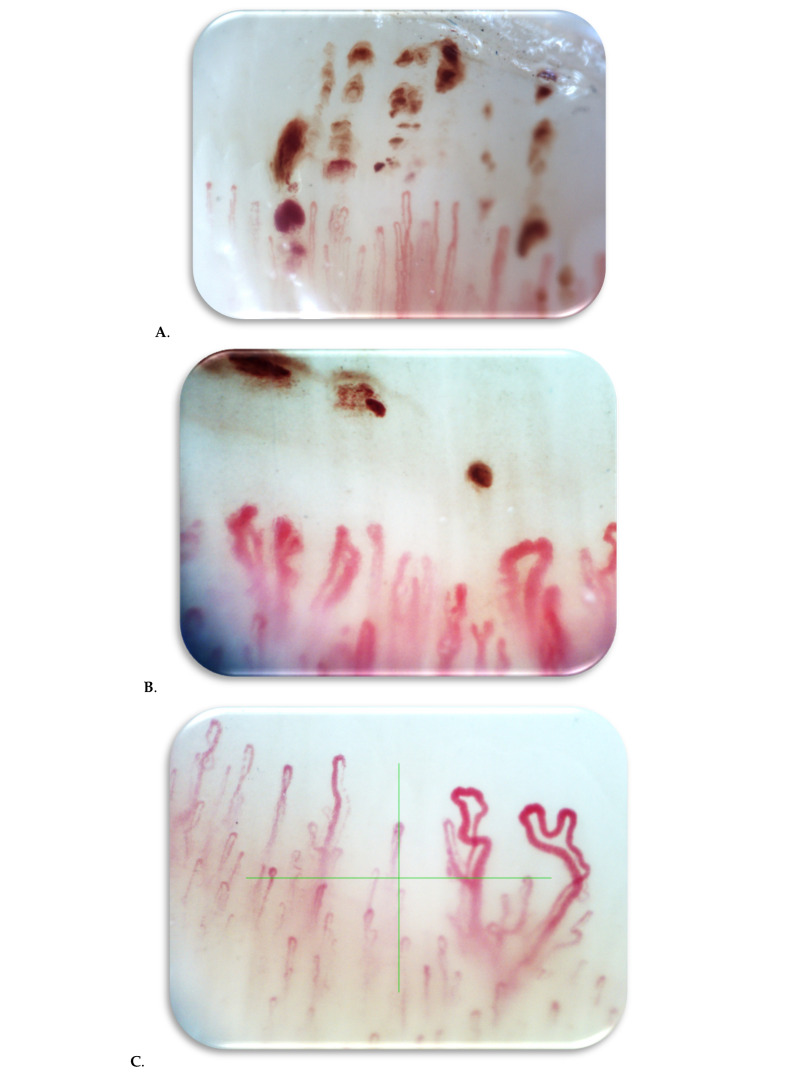
Jitter plot representation of NVC scores to visualize the distribution of individual one-dimensional values (density, giant, microhemorrhages, dilated capillaries, ramification, and disorganization). (**A**) Diffuse capillary microhemorrhages (×200). (**B**) Dilated capillaries >20 microns (×200), (**C**). Ramification (×200).

NVC scoring in AD and control groups is shown in Table 2. There were no differences in capillary density or giant capillaries between the AD group and the controls. The capillary density mean score was 0.0 with SD of 0.1 in the AD group and 0.0 in the control group. Statistically significant higher microhemorrhages (Figure 1A) were recorded in the AD group with a mean of 0.5 (SD 0.5) compared to controls with a mean of 0.1 (SD 0.1) and a *p*-value of 0.001. There were statistically significantly more enlarged capillaries present (Figure 1B) in the AD group compared to controls (0.5 with SD of 0.6 versus 0.1 with SD of 0.2, *p* < 0.001). There were also statistically significant increased ramifications (Figure 1C) in the AD group mean of 0.3 (SD 0.5) compared to the control group mean of 0.0 (SD 0.0). There were no differences in disorganized architecture between cases and controls.

The mean age (interquartile range [IQR] 53–72 years) in the AD group had a correlation with significant negative change in capillary ramification score on linear regression R = −0.02 (95% confidence interval [CI] −0.03, −0.006) (Table 1). Moreover, 12/27 (44%) in the AD group were females with no meaningful changes in NVC scores due to gender. The median BMI of the AD group was 26.6 (IQR 22.6, 30.9) with a positive correlation with capillary density [R = 0.005 (95% CI 0.001, 0.009)] and a negative correlation with dilated capillaries [R = −0.05 (95% CI −0.09, −0.01)] on linear regression (Table 1). Autonomic testing, including qsweat, cardiovagal responses, abnormal cardiovascular adrenergic response, CASS, and EMG, did not have any correlation with changes in NVC scores on linear regression.

## 4. Discussion

The presence of autonomic dysfunction and correlation with symptoms of autoimmune disorders has been most described in primary Sjogren’s syndrome (PSS) and less studied in other autoimmune connective tissue diseases [9]. Prior studies have investigated heart rate variability, autonomic reflex testing, impaired vasodilation, and gastric-emptying abnormalities strongly consistent with multiorgan autonomic dysfunction in PSS [10]. However, limited literature is available on the study of microvascular changes in patients with AD and has been described predominantly in systemic sclerosis (SSc), perhaps because of the role of NVC in the prognostication of SSc. Masini et al. demonstrated an abnormal NVC pattern in 23/26 (88.5%) SSc patients correlating with AD [11]. This is consistent with a study conducted by Franco et al. that also noted a correlation of the extent of NVC abnormalities with AD parameters in SSc, although autonomic testing was limited to heart rate variability [12].

The pathogenesis of AD in autoimmune disorders is largely unclear, although several different mechanisms have been contemplated. Impairment mediated by autoantibodies to nerves, vasculature, and tissue fibrosis are some possible explanations, with degeneration of neural networks and ganglion cells observed in SSc [13]. T-cell-mediated inflammation, cytokine-induced neuropeptide secretion, immune complex deposition, and pathogenic autoantibodies have been speculated to have a role in the development of autonomic dysfunctions in PSS [14]. In our study, only 7% of the patients had coexisting autoimmune disorders (Sjogren’s syndrome), indicating that NVC is a supportive diagnostic tool not exclusive to autoimmune rheumatic disorder diagnosis. It is important to note that the visualized NVC pattern was a non-scleroderma-like pattern, which further enhances the usefulness of NVC in this subset with its distinctive features.

We noted a distinct NVC phenotype in our AD subset, which included statistically significantly increased enlarged capillaries (>20 microns and <50 microns in diameter), microhemorrhages, and ramifications. There were no differences in giant capillaries (>50 microns in diameter), capillary density, and disorganized architecture in the AD and control groups. To our knowledge this is the first study that has established a discrete NVC pattern that correlates with the presence of AD. This can have significant implications for AD diagnostic tools and can encourage inculcation of microvasculature assessment, which is an objective measure easier to assess compared to non-specific subjective scoring tools that are currently available for AD testing. It is plausible that these findings correlate with the progression of AD; however, our study design was cross-sectional.

Unique to our study, an interesting observation made was a significant correlation with age and change in mean NVC ramification scores. Terreri et al. were the first to describe the effect of age on NVC findings [15]. Nakajima et al. recently noted a decrease in capillary length with aging and lifestyle factors such as body temperature, smoking, and stress correlating with NVC aberrations [16]. We are the first to identify a statistically significant direct correlation between BMI and microvascular changes, including a positive correlation with capillary density and a negative correlation with dilated capillaries. We noted a decrease in capillary density with an increase in BMI as well as a decrease in enlarged capillaries. Prior literature has reported changes in capillary density and dilations with BMI [17]. Repercussions of these correlations involve detailed modifiable risk factor assessment in patients with autonomic dysfunction and microvascular abnormalities and a potential role in AD management, which comes with its own challenges.

Strengths of our study include comprehensive autonomic testing, including comprehensive autonomic reflex testing, CASS scoring, assessment of flat-top response, and EMG. Also included were multiple various categories of AD ranging from POTS to central autonomic disorders, with all patients having abnormal autonomic reflex screening results. Our cases of autonomic dysfunction were reviewed by two neurology experts in autonomic dysfunction, which minimized selection bias. We also excluded other illnesses that affect the vasculature and may cause changes on capillaroscopy, including dialysis dependence, bleeding disorders, and recent trauma to the nailfold. A few limitations need to be taken into consideration when interpreting these results. The primary disadvantage is the sample size of our study. It may not have been adequately powered for generalizability of results. Other limitations of our study include a lack of blinding for the capillaroscopy procedure; however, this would have led to skewing of the results and overestimation of differences. We investigated the presence of skin nerve fiber density and salivary gland biopsy in cases with Sjogren’s syndrome and autoimmune dysautonomia panel. It is plausible that NVC aberrations correlate with the progression of AD; however, we did not delve into follow-up of our patients, which may have been clinically meaningful. Our study paves the way for future research to recognize the utility of NVC as a reliable and specific diagnostic tool that can aid in AD assessment as well as the identification of correlations between age and BMI that contribute to microvascular abnormalities.

## 5. Conclusions

Autonomic dysfunction was associated with statistically significant microvascular abnormalities compared to normal controls, including significantly increased enlarged capillaries, microhemorrhages, and ramifications. We demonstrated the diagnostic potential of NVC in autonomic dysfunction. Moreover, we advocate for further study of microvascular structure in autonomic dysfunction.

## Figures and Tables

**Table 1:** Single-variable linear regression exploring associations of characteristics with NVC scores. * means statistically significant.

		Estimated Difference in Mean NVC Scores (95% Confidence Interval)					
Factor	N (%) or Median (IQR)	Capillary Density	Giant Capillaries	Microhemorrhages	Dilated Capillaries	Ramification	Disorganization
Age	59 (53–72)	−0.002 (−0.014, 0.010)	NA	−0.01 (−0.02, 0.002)	−0.01 (−0.03, 0.0001)	−0.02 (−0.03, −0.006) *	NA
Female gender	12 (44%)	0 (−0.1, 0.1)	NA	0.1 (−0.3, 0.5)	0(−0.5, 0.5)	0.1 (−0.1, 0.3)	
BMI	26.6 (22.6, 30.9)	0.005 (0.001, 0.009) *	NA	−0.02 (−0.06, 0.02)	−0.05 (−0.09, −0.01) *	0.02 (−0.01, 0.06)	NA
Raynaud’s phenomenon	2 (7%)	NA	NA	NA (all 0 s in the 2 with disease)	0 (−1.0, 1.0)	−0.5 (−1.8, 0.8)	NA
Orthostatic hypotension	13 (48%)	0 (−0.1, 0.1)	NA	−0.2 (−0.6, 0.2)	−0.2 (−0.6, 0.2)	0.1 (−0.3, 0.5)	−0.1 (−0.3, 0.1)
Connective tissue disease diagnosis	2 (7%)	NA	NA	0 (−1.0, 1.0)	−0.8 (−1.2, −0.4)	0 (−0.6, 0.6)	NA
Autonomic dysfunction diagnosis (yes)	3 (11%)	NA	NA	−0.3 (−0.5, 0.1)	0.3 (−0.1, 0.7)	0.4 (−0.2, 0.6)	NA
Small-fiber neuropathy	10 (37%)	0 (−0.1, 0.1)	NA	0 (−0.3, 0.3)	0.2 (−0.3, 0.7)	0.3 (−0.1, 0.7)	NA
POTS	2 (7%)	0.1 (−0.2, 0.4)	NA	−0.1 (−0.8, 0.6)	−0.2 (−0.6, 0.2)	0.8 (0.2, 1.4)	0 (−0.3, 0.3)
Neuropathic POTS	0						
Limited autonomic neuropathy	2 (7%)	NA	NA	0.0 (−0.6, 0.6)	−0.4 (−0.7, 0.1)	0.3 (−1.0, 1.6)	NA
Autonomic neuropathy	8 (30%)	NA	NA	0.1 (−0.6, 0.4)	0.1 (−0.6, 0.4)	−0.2 (−0.6, 0.2)	0 (−0.2, 0.1)
Other central autonomic disorders	3 (11%)	NA	NA	−0.5 (−1.8, 0.8)	−0.8 (−1.4, −0.2)	0.3 (0, 0.6)	−0.5 (−1.5, 0.5)
MSA	0						
Familial dysautonomia							
Pure autonomic failure	0						
Autoimmune autonomic ganglionopathy	0						
Qsweat (reduced)	19 (70%)	0 (−0.1, 0.1)	NA	0.2 (−0.1, 0.5)	−0.3 (−0.7, 0.1)	0.2 (−0.3, 0.7)	0.2 (−0.1, 0.5)
Cardiovagal responses (reduced)	7 (26%)	NA	NA	−0.1 (−0.6, 0.4)	−1 (−0.6, 0.4)	0.3 (0, 0.6)	−0.1 (−0.4, 0.2)
Cardiovascular adrenergic responses	10 (38%)	NA	NA	0 (−0.4, 0.4)	−0.1 (−0.5, 0.3)	−0.2 (−0.5, 0.1)	0.1 (−0.1, 0.3)
Flat-top response							
Composite autonomic severity score (CASS)	3 (2, 5)	0 (−0.008, 0.006)	NA	−0.01 (−0.12, 0.11)	0.05 (−0.09, 0.19)	−0.09 (−0.19, 0.01)	NA
EMG	20 (77%)	NA	NA	0.1 (−0.3, 0.5)	−0.2 (−0.9, 0.5)	0.1 (−0.3, 0.5)	0 (−0.2, 0.2)
Skin punch biopsy	0						
ANA positivity	0						
SSA	0						
SSA−52+							
SSB	0						
Autoimmune dysautonomia panel	0						

**Table 2:** Overall comparison of NVC scores between groups.

Nailfold Videocapillaroscopy Scores	Autonomic Dysfunction (N = 27)	Controls (N = 21)	Total (N = 48)	*p* Value
Capillaroscopy density	0 (0.1)	0 (0)	0 (0)	--
Dilated capillaries	0.5 (0.6)	0.1 (0.2)	0.3 (0.5)	<0.001
Giant capillaries	0 (0)	0 (0)	0 (0)	--
Microhemorrhages	0.5 (0.5)	0.1 (0.1)	0.3 (0.4)	0.001
Ramification	0.3 (0.5)	0 (0)	0.10 (0.4)	<0.001
Disorganization	0.1 (0.2)	0 (0)	0 (0.2)	--
Mean (standard deviation) are shown for each group				

## Data Availability

The original contributions presented in this study are included in the article. Further inquiries can be directed to the corresponding author.
